# Phylogeography of the endangered “eyed” turtles (genus *Sacalia*) and the discovery of a lineage derived from natural interspecific hybridization

**DOI:** 10.1002/ece3.9545

**Published:** 2022-12-21

**Authors:** Liu Lin, Huai‐Qing Chen, Daniel Gaillard, Hai‐Tao Shi, Shu‐Jin Luo

**Affiliations:** ^1^ Ministry of Education Key Laboratory for Ecology of Tropical Islands, College of Life Sciences Hainan Normal University Haikou China; ^2^ The State Key Laboratory of Protein and Plant Gene Research, Peking‐Tsinghua Center for Life Sciences, School of Life Sciences Peking University Beijing China; ^3^ Center for Nature and Society, School of Life Sciences Peking University Beijing China; ^4^ School of Science, Technology, and Mathematics Dalton State College Dalton Georgia USA

**Keywords:** eyed turtles, interspecific hybridization, mitochondrial phylogeny, population genetic structure, *Sacalia bealei*, *Sacalia quadriocellata*

## Abstract

The herpetofauna of the Indomalayan bioregion of Asia suffers from severe habitat loss, unsustainable harvesting, and lack of research and conservation. Here, we investigated the range‐wide phylogeography of the endangered “eyed” turtles (genus *Sacalia*, including the Beale's Eyed Turtle *S. bealei* and the Four‐eyed Turtle *S. quadriocellata*) and discovered a natural interspecific hybrid turtle population in China. Based on phylogeny of the mitochondrial Cytochrome b gene of 101 samples in this study and public data, three major clades and six subclades were identified: *S. bealei* (SBE) in eastern‐southern China, east *S. quadriocellata* in South China (northern east [SQUen] and southern east [SQUes] subclades), and west *S. quadriocellata* mainly in Vietnam (northern west [SQUwn], central west [SQUwc], and southern west [SQUws] subclades). We sequenced 16 nuclear DNA loci of 87 samples from SBE, SQUen, SQUes, and SQUwn subclades. Population genetic clustering analysis suggested a structure similar to the mitochondrial phylogeny, where most samples were classified into four genetic clusters corresponding to the four mtDNA subclades. However, a proportion of samples carrying SQUen mtDNA haplotypes formed an additional distinct cluster SHY. Those samples are found in the contact zone of the two species bearing mosaic and intermediate morphological characteristics. We detected an admixed ancestry in SHY from SBE and SQUen that conformed to an intrapopulation breeding scenario for at least hundreds of generations after the initial hybrid event, leading to a conclusion that SHY is a distinct and near‐panmictic population derived from natural interspecific hybridization. In addition, SQUes (Hainan Island endemic) is of special concern due to significant isolation and low genetic diversity. We suggest that seven evolutionarily significant units should be recognized to facilitate appropriate conservation actions. These findings also highlight the urgent need for further herpetological research and conservation in this region.

## Graphical abstract

In this study, we investigated the phylogeography of the endangered “eyed” turtles (genus *Sacalia*) and gave a thorough grasp of lineage divergence, regional distribution, and gene flow in this genus' populations. We further confirm that the “hybrid eyed turtles” are a natural lineage developed from interspecific hybridization between *S. bealei* and *S. quadriocellata*. The conservation management of this genus will benefit greatly from these taxonomic and phylogenetic findings.

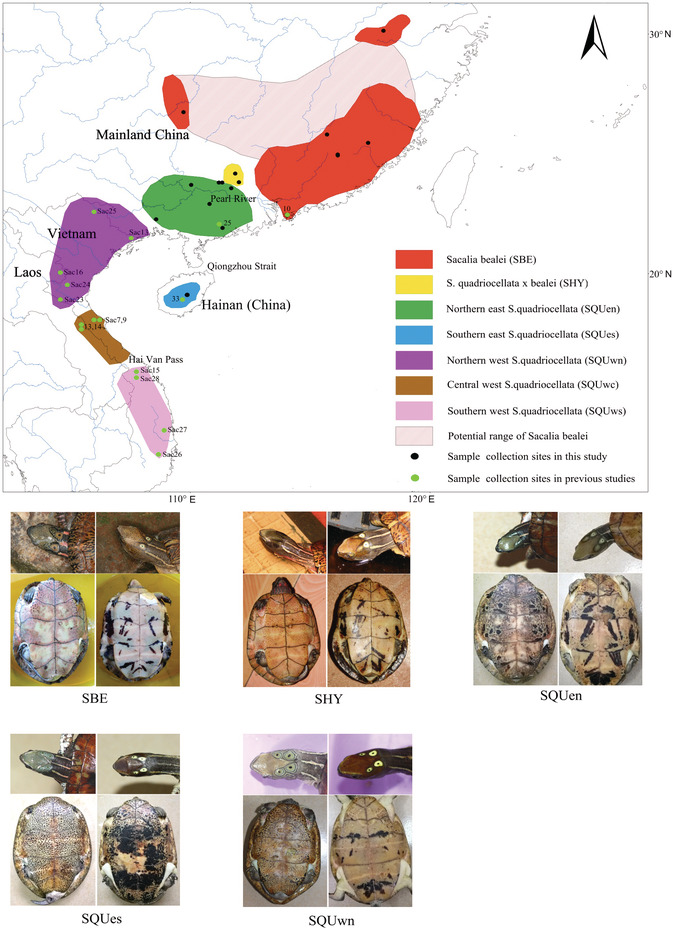

## 1 INTRODUCTION

The Indomalayan bioregion of Asia is one of the most important biodiversity hotspots worldwide, home to the extreme richness, and endemism of its turtle and tortoise species. This region encompasses over 89 species of chelonian fauna, or 25% of the world's total turtle diversity (Turtle Taxonomy Working Group, 2021). However, turtles in this region are most affected by anthropogenic effects, comprising over half (58%) of the top 50 most endangered turtles in the world (Turtle Conservation Coalition, 2018). This region is undergoing a severe crisis due to habitat loss and overexploitation of food, traditional medicine, and pet trade markets (Fong & Sung, 2017; Gong, Chow, et al., 2009; Gong, Shi, et al., 2009). The current status, diversity, and distribution of many Asian turtles are still poorly understood, requiring more in‐depth herpetological research, enhanced public awareness, and effective conservation (Wang et al., 2021).

**FIGURE 1:**
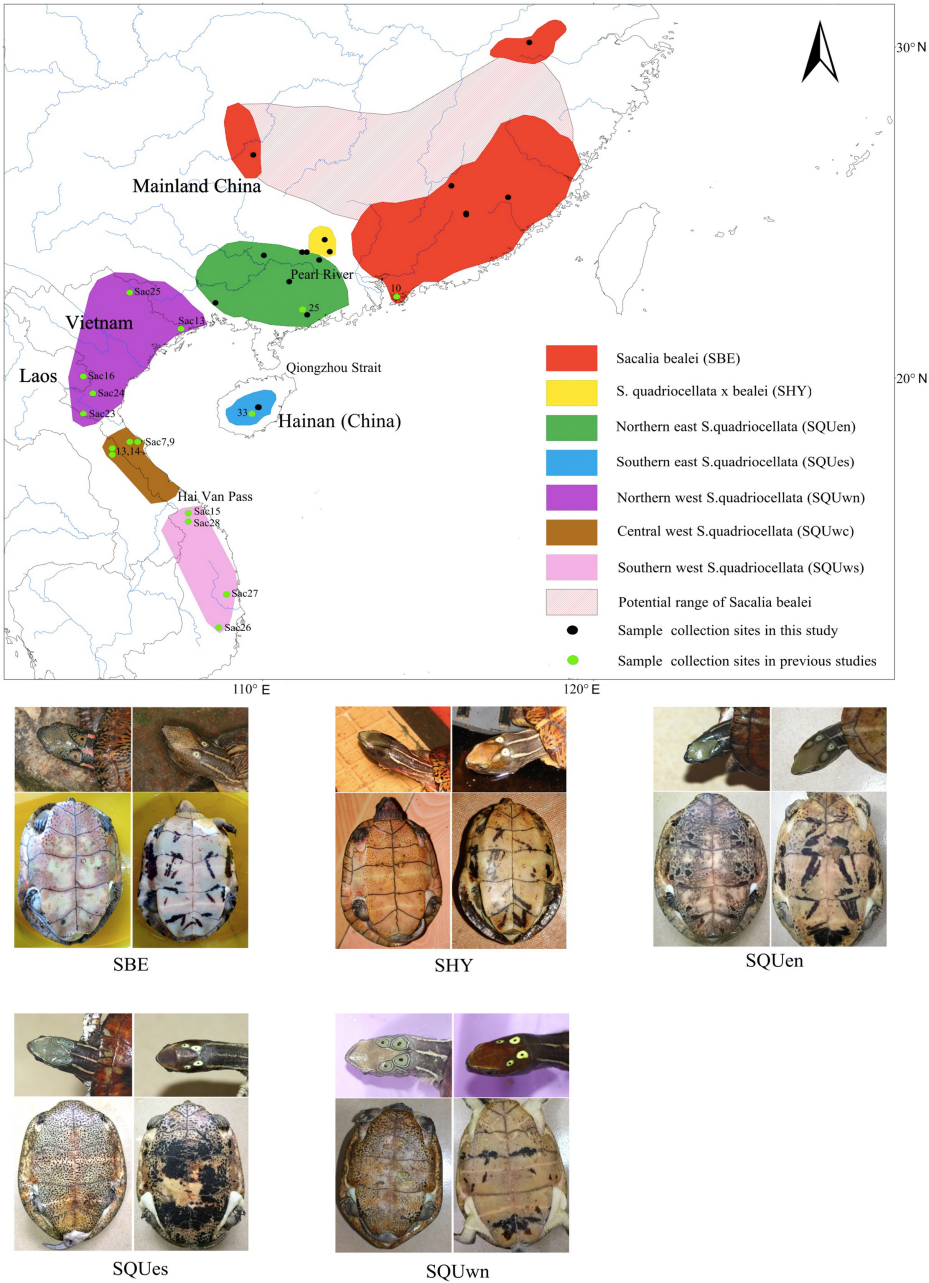
Map of geographic distribution, and morphological characters of eyed turtles *Sacalia* spp. The geographic range of each population is represented by colored blocks, and major river systems are presented. Black dots refer to the sample collection sites in this study; green dots refer to sites reported by Shi, Parham, et al. (2008); Shi, Fong, et al. (2008) and Le et al. (2020). Inserted pictures show morphological characteristics of the adult male (left) and female (right) from a specific population.

Turtles belonging to the genus *Sacalia* are distributed in southern China and northeastern Indochina, including two species with a range transition zone in Guangdong province, China: the Beale's Eyed Turtle *S. bealei* in the east and the Four‐eyed Turtle *S. quadriocellata* in the west (Figure 1a). Most noticeable for their eye‐like spots on the back of the head, the bright vertical stripes on the neck, and radial strips on the carapace, *Sacalia* turtles are increasingly popular among turtle hobbyists. Like many other turtle species, they are also consumed as food for their assumptive benefit to health and longevity (Fong & Sung, 2017). The market price of *Sacalia* turtles has been growing fast in recent years and reached $1000 per kg in 2016 (Hu et al., 2016). Both *Sacalia* species are now extremely rare in the wild (Gong et al., 2017) and listed as second‐class protected species on the “List of Wildlife under Special State Protection” in 2021 but are still open to commercial captive farming. Meanwhile, *S. quadriocellata* is listed as critically endangered, and *S. bealei* is endangered on the IUCN Red List of Threatened Species (IUCN, 2022).

The first mitochondrial phylogeographic study on *Sacalia* based on 1140 bp Cytochrome b (*CytB*) sequence, including known locality and market specimens, revealed a deep divergence between *S. bealei* and *S. quadriocellata* and identified three subclades (East, Northwest, Southwest) within *S. quadriocellata* (Shi, Fong, et al., 2008; Shi, Parham, et al., 2008). A recent phylogeographic study focusing on west *S. quadriocellata* further identified five subclades within Vietnam and Laos when more known‐locality samples were examined (Le et al., 2020). The *S. quadriocellata* population in Hainan Island, which was included in the east clade, showed significant morphological variance (Sun, 2015) and remarkable mitogenome divergence (Lin et al., 2020) from mainland populations. Thus, it was proposed as an independent species named *Sacalia insulensis* (Lin et al., 2018, 2020). However, since *Sacalia* turtles from mainland China with known locality were underrepresented in these studies, such sampling would be important to capture the full spectrum of phylogeographical diversity of the genus. In addition to mitochondrial phylogeography, nuclear gene‐based population genetic structure and morphological examination would provide better understanding of their evolution, taxonomy, and conservation.

Distinguishing cases of natural populations from anthropogenic hybridization is an important issue for the conservation of endangered wildlife species, particularly for those extensively bred for commercial purposes (Allendorf et al., 2001; Spinks et al., 2012). For *Sacalia*, a third taxon, “*S. pseudocellata*,” was once described as a new species from the pet trade, but was subsequently discarded as an F1 hybrid between *S. quadriocellata* and *Cuora trifasciata*, and hence considered invalid (Shi, Fong, et al., 2008; Shi, Parham, et al., 2008; Stuart & Parham, 2007). However, we recently discovered some wild *Sacalia* turtles with unique morphological features in the contact zone of *S. quadriocellata* and *S. bealei*. Although commonly called “hybrid‐eyed turtles” by pet keepers and traders in China, they may represent a novel, naturally occurring *Sacalia* population. A typical *S. quadriocellata* turtle has no or a few dots on the head, with two pairs of disjunct, bright eye‐like spots. An *S. bealei* turtle, by contrast, has dense black dots on its head and the two pairs of eye‐like spots are connected through the front pairs obscure (Figure 1b). These newly discovered “hybrid” turtles appear similar to *S. quadriocellata* at first glance but bear darker and nearly connected eye‐like spots, somewhat resembling *S. bealei*. Unlike the human‐mediated hybrid turtles “*S. pseudocellata*,” that were only found in captivity, the presence of these unusual *Sacalia* turtles has been confirmed in montane stream habitats quite similar to those of *S. quadriocellata* (Gong et al., 2006) and *S. bealei* (Hu et al., 2016).

Captive breeding of turtles for the pet market has become increasingly popular in China and the hybrids of turtles from different geographical regions or even from different species are common, which may threaten the genetic integrity of natural populations (Gong, Chow, et al., 2009; Gong, Shi, et al., 2009). On the other hand, a severe lack of understanding of the natural history, phylogeny, and morphological features of turtle species in this region may lead to the under‐representation of cryptic chelonian diversity in Asia (Gong et al., 2018). For example, for the softshell turtle genus *Pelodiscus*, three new species (*P. variegatus*, *P. huangshanensis*, and P. shipian) and one distinct lineage have been discovered in recent years, highlighting the underestimated diversity of *Pelodiscus* (Farkas et al., 2019; Gong et al., 2021, 2022).

In this study, we used a range‐wide sample dataset and applied both mitochondrial and nuclear DNA markers for the first time to elucidate the phylogeographic pattern and population structure of the entire *Sacalia* genus. We also used genetic ancestry analysis and simulation of hybridization to further investigate the origin of the potential hybrid *Sacalia* turtles. Based on the results, we provide recommendations for the taxonomy and conservation of *Sacalia* turtle species.

## 2 MATERIALS AND METHODS

### 2.1 Sampling and DNA extraction

Tissue and saliva samples were collected from 88 *Sacalia* turtles kept at Hainan Normal University or from local hobbyists or farmers in southern China (Table 1 and Table S1). Morphological identification keys, such as eye‐like spots and black dots on the head, and spots or patches on the plastron, were used for a preliminary species classification for each individual as a Beale's Eyed Turtle (*n* = 16), Four‐eyed Turtle (*n* = 47), or a potential “hybrid” (*n* = 25) (Shi et al., 2013). We carefully conducted interviews with owners to ensure that no sampled individual was bred in captivity and the geographic source of each turtle was recorded to the best of our knowledge. We recorded one sample of the hybrid‐eyed turtle with exact locality source in the field survey, and information from multiple local dealers confirmed that the “hybrid” eyed turtles were only collected from a few adjacent areas of the known locality. Afterward, saliva samples of nine *S. bealei* and five *S.quadriocellata* were added to fully disclose the mitochondrial phylogeny, but nuclear markers from these additional samples were not analyzed (Table 1 and Table S1).

**TABLE 1:** *Sacalia* spp. samples used in this study.

Taxa	Subclade/population	Geographic region	Specific locality	Sample size
Beale's Eyed Turtle	SBE (*N* = 25)	East Guangdong	Meizhou	3
(*S. bealei*)		Fujian	Zhangping	4
		Hunan	Suining	6
		Anhui	Xuangcheng	9
		Jiangxi	Huichang	3
Four‐eyed Turtle	SQUen (*N* = 24)	West Guangdong	Yangjiang	3
(*S. quadriocellata*)			Zhaoqing	7
		East Guangxi	Wuzhou	9
		Unclear		5
	SQUes (*N* = 8)	Hainan	Hainan	8
	SQUwn (*N* = 11)	Unclear		11
	SQUwc (*N* = 2)	Unclear		2
	SQUws (*N* = 4)	Unclear		4
Hybrid Eyed Turtle	SHY (*N* = 27)	East Guangxi	Hezhou	1
(*S. quadriocellata × bealei*)			Wuzhou	3
		West Guangdong	Zhaoqing	23
Total				101

Abbreviations: SBE, *S. bealei*; SHY, *S. quadriocellata × bealei*; SQUen, northern east *S.quadriocellata*; SQUwn, northern west *S. quadriocellata*; SQUes, southern east *S. quadriocellata* or Hainan *S. quadriocellata*; SQUwc, central west *S. quadriocellata*; SQUws, southern west *S. quadriocellata*.

Saliva samples were collected using a PERFORMAgene PG‐100 swab kit (DNA Genotek, Ottawa, Ontario, Canada) or Hi‐Swab DNA Kit (Tiangen, #DP3622, Beijing, China). If a buccal swab could not be obtained, a cloacal swab was collected. If permitted by the owners, approximately 2 mm of tissue was cut from the tail tip and preserved in 95% ethanol. Genomic DNA from tissues and swab samples were extracted with a DNeasy Blood & Tissue Extraction Kit (QIAGEN, Valencia, California, USA) following the manufacturer's protocols.

The study protocol was approved by the Animal Research Ethics Committee of Hainan Provincial Education Center for Ecology and Environment, Hainan Normal University (No. HNECEE‐2014‐003).

### 2.2 Genetic markers, PCR amplification, and sequencing

Phylogenetic and population genetic analyses were conducted using the mitochondrial *CytB* and 16 nuclear genes (Table 2). Two overlapping mtDNA amplicons were designed based on reference sequences (GenBank accession numbers GU320209, NC_011819, GU183364) to cover the full length of *CytB* (1144 base pairs [bp]). To screen for nuclear genetic diversity, 50 markers were selected for pilot test from a set of 104 loci previously applied for the Western Pond Turtle (*Actinemys marmorata*; Spinks et al., 2014), under the criteria that the amplified fragment was no larger than 800 bp, contained 3–8 variable sites, and had no insertion‐deletions (indels). We sequenced the 50 loci in a pilot five‐sample set, and 16 markers, which included at least two variable sites and no more than two indels, were selected as the final panel to be applied for all individuals (amplicon length 420–800 bp, Table S2). PCR primers for two loci (TB73 and VIM) were redesigned for optimized amplification using Primer 3 (Untergasser et al., 2012).

**TABLE 2:** PCR amplicons for mtDNA and 16 nuclear markers used in this study.

Primer name	Forward primer (5′‐3′)	Reverse primer (5′‐3′)	Fragment size/bp	Clean data size/bp	Original literature
*Mitochondrial DNA markers*					
Sca_cytb_1f/1r_new	ACCAAGACCTGTGATTTGAAAA	AGGTGAGTGTTAGGATGAGGC	768	1144	This study
Sca_cytb_2f_new/2r	CAGTAGACAACGCCACCCTA	TGTGGTCTTCAGTCTTTGGTTT	694		This study
*Nuclear DNA markers*					
17367	GGAGTCCTGATGGATGACAC	GGTGGCTTTATACCCATCTC	800	589	Backstrom (2008)
AKR	GTCTGCAACTGGTTTATCAAC	TTAGGCCATGAGTTTTGCCTG	529	423	Berlin et al. (2008)
DNAH3	GGTAAAATGATAGAAGAYTACTG	CTKGAGTTRGAHACAATKATGCCAT	800	625	Townsend et al. (2008)
FSHR	ATATGGTTTATCARCATTYTAGC	CTTGGATTTGGASACWGTRATGAG	668	652	Spinks et al. (2014), Townsend et al. (2008)
GHR	TGATGATTCTGGACGKGCCAGTTG	TTGAGCACAAGGCCTTGTGGAG	617	605	Spinks et al. (2014)
NB14108, DDAH1	TCCAAGCGGACAAATCAACG	GGGCTTTCTGTGCAGCTTC	734	640	Backström et al. (2008)
PLXNA2	AGGAYAACAARTCMTGCTACC	TTRGTGCACTGSACRTCCTTYCC	770	688	Spinks et al. (2014)
PRLR	GACARYGARGACCAGCAACTRATGCC	GACYTTGTGRACTTCYACRTAATCCAT	532	445	Townsend et al. (2008)
TAR	TTGCCCAGTCTCTTTGTGGAG	CTACATTCCCCAGCCTGA	503	491	Berlin et al. (2008)
TB29	GGTACCAAGCATACCCATTTG	GGTTCAATAAGAATGGGGAAGA	609	490	Thomson et al. (2008)
TB54	CAAGCTGTTGTCTATGGAGTACTTTC	CCTGCTATTGAATGACATATACTGC	714	584	Thomson et al. (2008)
TB62	AACTTGCTGACTGACGTAAGAAAA	GGAGTTATCTTGTTTGAAGTTAAAGG	703	580	Thomson et al. (2008)
TB73_new	CCTGCAATGGGGTCTGCTA	TGCAAAATCTACTCCAGCTTAGG	420	376	This study, Thomson et al. (2008)
TB75	AAATGTTGCGTTGACAATTCAG	GTGGCTGGCTTTTTGTAGGT	608	460	Thomson et al. (2008)
TB81	AGGCTCTCTTCTGCCATTCA	GAGCCAAAATTTTTCCTTTGC	745	618	Thomson et al. (2008)
VIM_new	AGTGGAGGAGGAAGTAAAACAAA	ACCTGTTGAAGAATAAGACAGCT	633	523	This study, Kimball et al. (2009)

All mitochondrial and nuclear loci were amplified in a 15 μl PCR reaction system containing 1× PCR buffer II, 0.8 mM dNTPs, 2 mM MgCl_2_, 0.5 mg/ml BSA, 0.75 units of AmpliTaq Gold DNA polymerase (Applied Biosystems), 0.25 μM of forward and reverse primer, and ~10 ng of DNA. The PCR procedure consisted of an initial denaturation at 95°C for 10 min, touchdown cycles of denaturation at 95°C for 15 s, annealing at 60–50°C (decreased by 2°C every two cycles) for 30 s, and extension at 72°C for 45 s, followed by 35 amplification cycles of 95°C for 15 s, 50°C for 30 s, and 72°C for 45 s, with a final extension of 10 min at 72°C. PCR products were purified with 1.9 units of Exonuclease I (ExoI, GE Healthcare Ltd.) and 0.37 units of shrimp alkaline phosphatase (SAP, GE Healthcare Ltd.) in a 10 μl reaction at 37°C for 15 min and denatured at 80°C for 15 min. Amplicons were sequenced with both forward and reverse primers separately on an ABI 3730XL sequencing system (Applied Biosystems).

Sequences were assembled and corrected using Sequencher 5.1 (Gene Codes Corporation, Ann Arbor, MI, USA). Nuclear sequences with two or more indels within the same amplicon, which could not be differentiated via direct Sanger sequencing, were discarded. The alleles of each nuclear locus were reconstructed with PHASE2.1.1 (Stephens et al., 2001; Stephens & Donnelly, 2003), and those with a posterior probability of <95% were discarded following previously described procedures (Spinks et al., 2012). A PERL script was developed to identify haplotypes for each locus and the genotype for each individual.

### 2.3 mtDNA phylogeny reconstruction

MtDNA haplotypes in this study, together with reference sequences of specimens with known geographic localities described in Le et al. (2020) and Shi, Parham, et al. (2008); Shi, Fong, et al. (2008), as well as those of two closely related species *Heosemys grandis* and *Cyclemys dentata* (GenBank accession numbers # JN582334 and # KX816868), were aligned using MEGA 6.0 (Tamura et al., 2013).

Mitochondrial phylogeny was reconstructed using maximum likelihood (ML) (with PAUP* 4.0b10; Swofford, 2003) and Bayesian inference (with MrBayes v3.2.2; Ronquist et al., 2012) approaches. Substitution models were tested using jModelTest 2.1.4 (Darriba et al., 2012), and HKY + G [Base = (0.3123 0.3136 0.1050) Nst = 2 tratio = 8.4601 Rates = gamma Shape = 0.2560 Ncat = 4] was selected as the best‐fit model under the Bayesian information criterion and applied to ML analyses. Bootstrapping of 100 replicates was conducted using the same parameters for tree construction with a heuristic search for ML. Bayesian inference was performed with three independent runs, each comprising four incrementally heated chains that ran for 11,000,000 generations under the substitution model of HKY + G. Trees and posterior distribution were sampled every 1000 generations, and the first 10% generations were discarded as burn‐in.

### 2.4 Nuclear DNA (nuDNA) genetic analysis

NuDNA genotypes of each individual were coded using a two‐digit numerical system representing the phased information at each locus across the 16 loci and were used as the input for Bayesian clustering analysis using STRUCTURE v2.3 (Pritchard et al., 2000). MCMC simulations were run with 1,000,000 replicates and a burn‐in of the first 10% replicates under an admixed genetic ancestry model without prior population source information. Each run was repeated 30 times, and the first ten with the highest likelihood ratio were saved for subsequent analysis. The number of clusters (K) was set from two to eight, and the likelihood of *K* was evaluated using the online tool STRUCTURE HARVESTER (Earl & von Holdt, 2012). CLUMPP (Jakobsson & Rosenberg, 2007) was used to combine the best ten replicates, and DISTRUCT (Rosenberg, 2004) was used to plot the results. The input for STRUCTURE v2.3 was also transformed to the “genind” format using the R package “adegenet” (Jombart, 2008; Jombart & Ahmed, 2011) and principal component analysis (PCA) was performed using the prcomp function of the R package “stats” and plotted using the R package “ggplot2.”

Splitstree 4.0 (Huson & Bryant, 2006) was used to reconstruct a genetic distance network based on concatenated non‐phased sequences from 16 nuclear DNA markers. Ambiguous sites were treated as average states and normalized. Uncorrected p‐distance and NeighborNet methods were used and the p‐distance matrix produced was plotted as a heatmap using the R package “pheatmap.” Isolation by distance was tested by the correlation of individual pair‐wise geographic distance and the genetic distance of samples with clear locality, which was plotted using the R package “ggpubr.”

### 2.5 Statistics of genetic diversity

Individuals with a primary nuclear DNA affiliation to a certain cluster (*Q* value) by more than 0.85 in STRUCTURE were used to represent each of the five clusters for population genetics statistics. Observed and expected heterozygosity and Hardy–Weinberg equilibrium for each marker were evaluated using the R package “adegenet.” Pair‐wise fixation index (*F*_ST_) was calculated using the R package “hierfstat” (Goudet, 2010) with the “pairwise.neifst” function, and a 95% confidence interval of 1000 replicates of the bootstrap test was performed with the “boot.ppfst” function. The marker VIM_new was excluded to reduce the missing data rate. The *F*_ST_ matrix was plotted as a network using the R package “phangorn” (Schliep, 2011).

### 2.6 Genetic ancestry analysis and simulation of hybridization

To elucidate the possible genetic origin of the putative natural hybrid lineage, we phased a subset of single nucleotide polymorphisms (SNP) from sequences of the 16 nuclear markers. Considering that total 60 individuals were used to represent the three populations SQUen (*N* = 18), SBE (*N* = 16), and SHY (*N* = 26), rare SNP which occurred <20 times were discarded to reduce number of alleles. Allele frequencies were plotted as a heatmap using the R package “pheatmap,” and pair‐wise *F*_ST_ between the two possible ancestral lineages SBE and SQUen was calculated using the R package “hierfstat.”

Based on eight species‐diagnostic loci distinguishing SBE and SQUen, the genetic composition of SHY was determined using the R package “introgress” (Gompert & Alex, 2010). To recapitulate the pattern of post‐hybridization genetic drift, genotypes of the first generation of interspecific hybridization and subsequent 999 generations of intrapopulation breeding were simulated using the R package “adegenet,” with SBE and SQUen genotype data set as the two parental populations. The population size was set at 100 individuals for each generation. Four independent replicates were used. The genetic makeup of the 1st, 2nd, 5th, 10th, 20th, 50th, 100th, 200th, and 500th generations was estimated and plotted using the R package “introgress.”

## 3 RESULTS

### 3.1 mtDNA phylogeny

**FIGURE 2:**
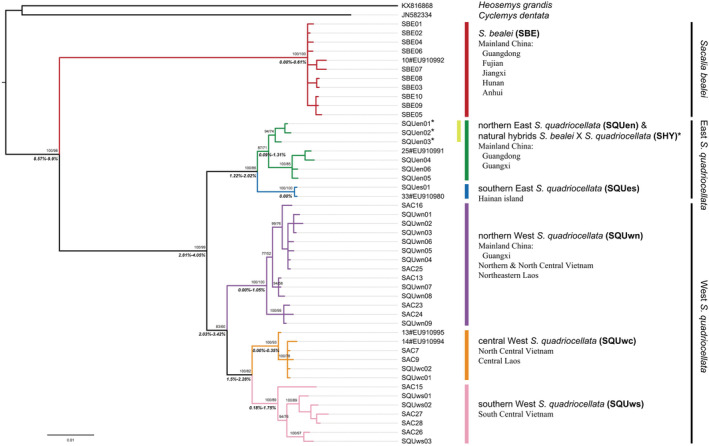
Phylogenetic tree of the genus *Sacalia* with the 1144 bp complete mitochondrial *CytB*. The posterior probability for Bayesian inference and bootstrap values for maximum likelihood methods are labeled next to the corresponding major branches. The branches linked to outgroups are truncated for a clearer view. Reference sequences of voucher samples from Shi, Parham, et al. (2008); Shi, Fong, et al. (2008) and Le et al. (2020) are marked with “#” or “SAC.” Three major clades and six subclades were identified. The haplotype codes of our samples are listed at end nodes, which are corresponding to Table S1. The three clades and six subclades are indicated by the bar on the right. Haplotypes shared by SHY from SQUen subclade are marked by asterisks.

Of the 102 *Sacalia* spp. samples, 101 were amplified and sequenced for 1144 bp mitochondrial *CytB*. Only one Four‐eyed Turtle cloacal swab sample (HBET0050) failed to yield adequate DNA for downstream analysis (Table S1). A total of 31 haplotypes were identified (Table S1), forming two deeply divergent clades in the phylogenetic tree (Figure 2). One clade, namely SBE, contained exclusively *S. bealei* and the other included all voucher specimens of *S. quadriocellata* and all putative hybrid turtles. Within *S. quadriocellata*, two major clades, east and west *S. quadriocellata*, were found, consistent with the pattern reported by Le et al. (2020) and Shi, Parham, et al. (2008); Shi, Fong, et al. (2008), and five subclades were further specified. The uncorrected genetic distance was 8.57%–9.9% between *S. bealei* and *S. quadriocellata*, 2.81%–4.05% between the two clades of *S. quadriocellata*, 1.22%–3.42% between subclades in the same clade, and 0–1.75% within each specific subclade.

*Sacalia bealei* represented a panmictic population and endemic in mainland China, including eastern Guangdong, Hong Kong, Fujian, southern Jiangxi, and southern Hunan. Two isolated populations of *S. bealei* are located in Anhui and a small area bordering Hunan and Guizhou, respectively. East *S. quadriocellata* consisted of two subclades, the northern east subclade (SQUen), including almost all specimens from Guangdong and Guangxi in mainland China, and the southern east subclade (SQUes) endemic to Hainan Island (Figure 1). West *S. quadriocellata* was distributed in Vietnam and bordering Laos and Guangxi, China, which was more specifically divided into northern (SQUwn), central (SQUwc), and southern (SQUws) subclades. SQUwn corresponded to the northwest subclade in Shi, Parham, et al. (2008); Shi, Fong, et al. (2008) and Clade B in Le et al. (2020), ranging from northern Vietnam and maybe west Guangxi, China. SQUwc and SQUws corresponded to the southwest subclade in Shi, Parham, et al. (2008); Shi, Fong, et al. (2008) and Clade A in Le et al. (2020), while SQUwc ranged from north‐central Vietnam and central Laos, and SQUws ranged from central Vietnam and south‐central Vietnam. All suspected hybrids shared three closely related mtDNA haplotypes in the subclade SQUen and were distributed in a restricted contact zone of Guangdong and Guangxi where SQUen and SBE met.

### 3.2 nuDNA population structure analysis

Amplification of the 16 nuclear markers generated a total of 8.7 kb of DNA sequences for most of the 87 samples, except for failed PCR amplification and unreadable multiple indels (Table S3), and genotyping for nuclear loci had an average of 7.1% missing data, ranging from 1.1% to 13.8% for each locus, except for VIM_new, which was unusually high at 25.3% (Table 3). To make sure no bias from the missing data of VIM_new, we tested dataset without VIM_new in STRUCTURE analysis, and the result was nearly the same with negligible variation of *Q* score. Additionally, the document of Bayesian STRUCTURE software states that there is no particular reason to exclude individuals with missing data. Therefore, we believe the dataset is unbiased, and it is not necessary to recover missing data with extra methods. Our 87 samples were obtained from four mitochondrial subclades (SBE, SQUen, SQUes, and SQUwn), but not SQUwc and SQUws. Therefore, our population genetics analysis only involved the four subclades and the hybrid population.

**TABLE 3:** Statistics of genetic diversity in *Sacalia* spp.

Marker name	All					SBE					SHY				
	Sample size after PHASE^a^	Number of alleles	He	Ho	*p* Value**	Sample size after PHASE^a^	Number of alleles	He	Ho	*p* Value**	Sample size after PHASE^a^	Number of alleles	He	Ho	*p* Value**
17367	84 (3.4%)	26	0.909	0.619	**.000**	16	9	0.754	0.813	.245	25	10	0.818	0.760	.210
AKR	86 (1.1%)	7	0.621	0.430	**.000**	16	3	0.537	0.500	.399	25	4	0.498	0.640	.130
DNAH3	75 (13.8%)	13	0.581	0.347	**.000**	16	3	0.389	0.500	.623	21	5	0.334	0.286	**.019**
FSHR	78 (10.3%)	9	0.590	0.295	**.000**	16	3	0.320	0.250	.211	25	5	0.315	0.240	**.022**
GHR	83 (4.6%)	9	0.649	0.361	**.000**	16	3	0.564	0.438	**.004**	26	4	0.473	0.346	.223
NB14108	79 (9.2%)	12	0.615	0.456	**.000**	14	3	0.625	0.714	.418	26	3	0.292	0.231	.063
PLXNA2	75 (13.8%)	18	0.864	0.533	**.000**	15	5	0.656	0.467	**.044**	23	7	0.346	0.261	**.048**
PRLR	86 (1.1%)	11	0.728	0.453	**.000**	16	7	0.752	0.563	.090	25	7	0.601	0.600	.616
TAR	85 (2.3%)	8	0.621	0.376	**.000**	16	3	0.648	0.563	.790	26	3	0.510	0.346	.093
TB29	80 (8.0%)	17	0.735	0.475	**.000**	16	3	0.461	0.500	1.000	26	5	0.466	0.308	**.038**
TB54	82 (5.7%)	9	0.456	0.354	**.000**	14	2	0.133	0.143	1.000	25	3	0.525	0.640	.112
TB62	84 (3.4%)	11	0.739	0.369	**.000**	15	4	0.747	0.467	.096	26	4	0.589	0.346	**.000**
TB73_new	83 (4.6%)	17	0.843	0.458	**.000**	16	6	0.717	0.188	**.000**	26	7	0.545	0.538	.382
TB75	85 (2.3%)	16	0.730	0.447	**.000**	15	6	0.798	0.533	.059	26	5	0.467	0.385	.269
TB81	79 (9.2%)	17	0.689	0.430	**.000**	15	6	0.662	0.600	.151	25	6	0.374	0.240	.005
VIM_new	65 (25.3%)	20	0.839	0.431	**.000**	14	5	0.462	0.500	.496	22	6	0.473	0.273	.005

Marker name	SQUen					SQUwn					SQUes				
	Sample size after PHASE	Number of alleles	He	Ho	*p* Value**	Sample size after PHASE	Number of alleles	He	Ho	*p* Value**	Sample size after PHASE	Number of alleles	He	Ho	*p* Value**
17367	18	8	0.733	0.444	**.000**	6	5	0.736	0.667	.236	8	1	0.000	0.000	1.000
AKR	18	3	0.625	0.389	**.000**	7	2	0.337	0.143	.213	8	2	0.117	0.125	1.000
DNAH3	14	5	0.370	0.286	.075	7	5	0.704	0.714	1.000	7	2	0.133	0.143	1.000
FSHR	15	2	0.231	0.267	1.000	4	3	0.406	0.250	.132	8	1	0.000	0.000	1.000
GHR	16	5	0.420	0.438	.462	7	3	0.520	0.429	.309	8	1	0.000	0.000	1.000
NB14108	16	8	0.803	0.563	.140	7	6	0.786	0.571	.227	7	2	0.133	0.143	1.000
PLXNA2	16	9	0.844	0.813	.601	7	6	0.786	0.714	.663	7	3	0.663	0.714	.365
PRLR	18	3	0.369	0.389	.627	7	3	0.255	0.286	1.000	8	2	0.375	0.250	.380
TAR	18	5	0.336	0.278	.488	7	5	0.776	0.857	.725	8	1	0.000	0.000	1.000
TB29	13	8	0.754	0.769	.131	7	3	0.582	0.429	.131	8	2	0.219	0.250	1.000
TB54	16	6	0.564	0.375	.082	7	1	0.000	0.000	1.000	8	1	0.000	0.000	1.000
TB62	17	5	0.502	0.529	.883	7	1	0.000	0.000	1.000	8	2	0.117	0.125	1.000
TB73_new	17	8	0.798	0.647	.135	6	4	0.639	0.667	1.000	8	4	0.555	0.250	.039
TB75	18	8	0.707	0.722	.819	7	3	0.255	0.286	1.000	8	1	0.000	0.000	1.000
TB81	18	4	0.366	0.444	1.000	7	5	0.622	0.571	.311	3	4	0.667	0.667	.603
VIM_new	12	12	0.906	0.833	.280	4	4	0.563	0.500	.433	7	1	0.000	0.000	1.000

Abbreviations: SBE, *S. bealei*; SHY, *S. quadriocellata × bealei*; SQUen, northern east *S. quadriocellata*; SQUwn, northern west *S. quadriocellata*; SQUes, southern east *S. quadriocellata* or Hainan *S. quadriocellata*; SQUwc, central west *S. quadriocellata*; SQUws, southern west *S. quadriocellata*.

** *χ* ^2^ test for Hardy–Weinberg equilibrium, markers significantly rejected (*p* < .05) are marked in bold.

a Missing data rates are in brackets.

**FIGURE 3:**
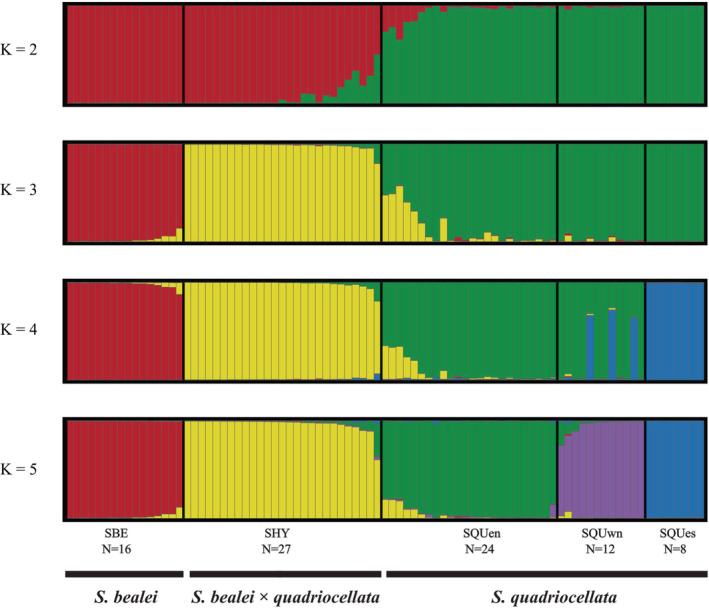
Bayesian population structure analysis‐based phased genotypes of 16 nuclear markers. The number of assumed clusters is from 2 to 5, labeled on the left. Each column represents one individual, assigned to one of the five populations according to their *Q* value when *K* is 5.

STRUCTURE analysis (Figure 3) suggested five clusters of populations. When *K* was 5, the increase in △*K* in STRUCTURE HARVESTER reached the highest value, and no further subdivision could be identified as *K* increased. The five clusters corresponded to SBE, SQUen, SQUes, SQUwn, and the suspected hybrids (SHY). When *K* was 3, the three clusters corresponded to SBE, SHY, and the three *S. quadriocellata* subclades, manifesting SHY as a distinct panmixia. Although each individual was assigned to a cluster according to the highest *Q* value, seven individuals (four in SQUen, two in SQUwn, and one in SHY) had *Q* values <0.85, which indicated the offspring of initial generations of interpopulation mating (Tables S1 and S5). The mito‐nuclear discordance, in which three individuals carried mtDNA haplotypes of SQUwn (haplotype SQUwn04 and SQUwn07, see Table S1) were assigned to SQUen, provided another genetic introgression evidence between SQUen and SQUwn. In further substructure analysis of SQUen and SQUwn, it was revealed that two individuals in SQUen and three individuals in SQUwn formed two more subdivisions (Table S1 and Figure S1).

**FIGURE 4:**
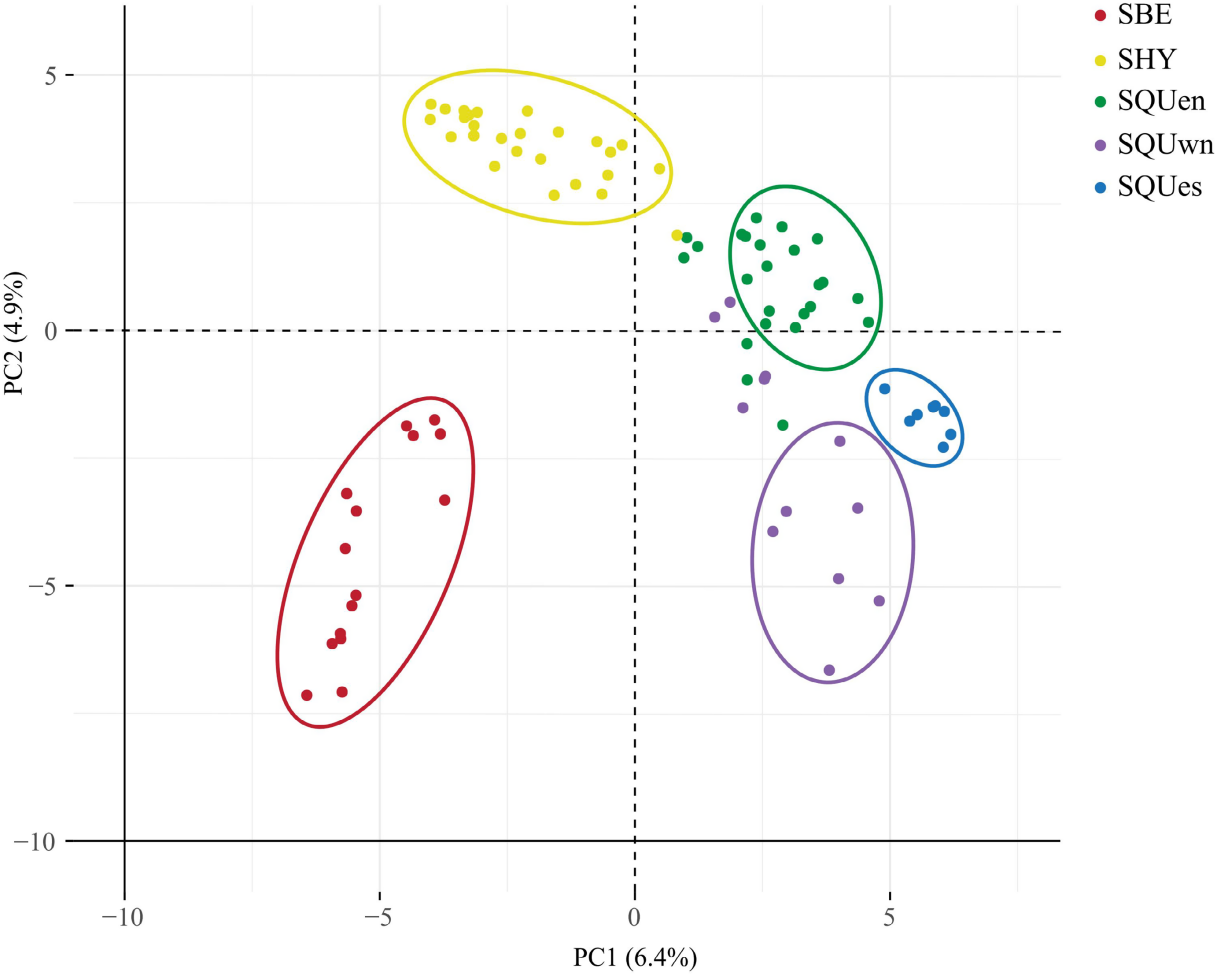
Principal component analysis (PCA) with 16 nuclear DNA markers. Five populations were grouped with the first two principal components, represented using different colors. Individuals with *Q* value >0.85 in Bayesian population structure analysis are grouped in circles, while those with *Q* value <0.85 are outside the circles, as well as two individuals in SQUen and three individuals in SQUwn with substructures.

The PCA results (Figure 4 and Figure S2) were concordant with the STRUCTURE results, in which samples were mainly separated into five clusters under the first three principal components. However, unlike the clear‐cut clusters of SBE and SQUes, 12 individuals from SHY, SQUen, and SQUwn were located in the intermediate region, which were the same individuals with a *Q* value <0.85 or which formed a substructure in the STRUCTURE analysis. The substructure is highlighted in minor principal components.

### 3.3 Genetic diversity and distance

A total 75 individuals were used to represent the five populations SBE (*N* = 16), SQUen (*N* = 18), SQUwn (*N* = 7), SQUes (*N* = 8), and SHY (*N* = 26), which has a *Q* score more than 0.85 in STRUCTURE analysis and are not in substructure. Most markers in the five populations adhered to the Hardy–Weinberg equilibrium (Table 3 and Figure S4). Pair‐wise *F*_ST_ is 0.332–0.534 between the island population (SQUes) and mainland populations (SQUen, SQUwn, SBE, and SHY), significantly higher than that among mainland populations (0.147–0.297), indicating that SQUes was mostly isolated (Table 4 and Figure S5).

**TABLE 4:** Pair‐wise *F*_ST_ values (below diagonal) and corresponding confidence intervals (95%; 1000 bootstrap; above diagonal) among the five *Sacalia* populations based on 15 nuclear DNA markers.

	SBE	SHY	SQUen	SQUwn	SQUes
SBE		0.148–0.289	0.214–0.339	0.191–0.307	0.392–0.530
SHY	0.215		0.189–0.321	0.249–0.369	0.431–0.543
SQUen	0.273	0.249		0.098–0.203	0.207–0.365
SQUwn	0.250	0.297	0.147		0.252–0.480
SQUes	0.500	0.534	0.332	0.386	

Abbreviations: SBE, *S. bealei*; SHY, *S. quadriocellata × bealei*; SQUen, northern east *S. quadriocellata*; SQUwn, northern west *S. quadriocellata*; SQUes, southern east *S. quadriocellata* or Hainan *S. quadriocellata*; SQUwc, central west *S. quadriocellata*; SQUws, southern west *S. quadriocellata*.

**FIGURE 5:**
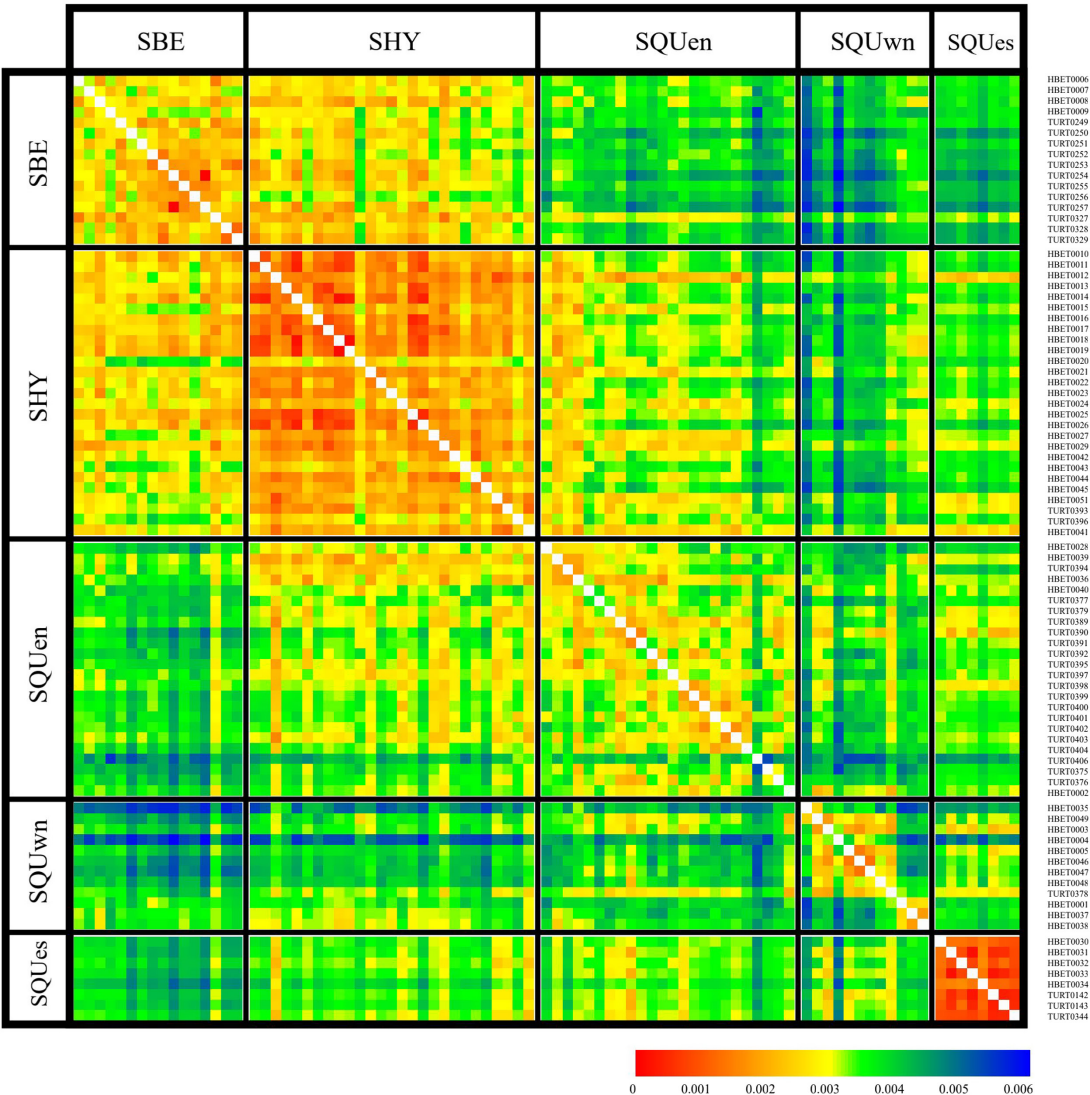
Heatmap of the p‐distance for 16 nuclear markers. The p‐distance matrix was produced using Splitstree 4.0 with the NeighborNet method and plotted as a heatmap using the R package “pheatmap.” The ambiguous sites were treated as average states and normalized.

We tested two hypotheses of the genetic ancestry of SHY: H1, SHY is an isolated population derived from its parental mitochondrial subclade SQUen; H2, SHY is hybrids between SQUen and SBE. Isolation by distance was detected within each of the three populations but not between them (Figure S8), which indicates the genetic uniqueness of SHY is not a result of isolation by distance (rejecting H1). The average p‐distance between SHY and SBE or SQUen was 0.0028 and 0.0030, respectively, notably lower than that between SBE and SQUen (0.0038) (Figure 5). *F*_ST_ between SHY and SBE (0.215; 95% ci: 0.148–0.289) and between SHY and SQUen (0.249; 95% ci 0.189–0.321) are also smaller than that between SQUen and SBE (0.273; 95% ci: 0.214–0.339). The pattern of p‐distance and *F*_ST_ conformed to that SHY has admixed ancestry from SBE and SQUen (supporting H2). In summary, our results indicate that SHY is a panmixia derived from hybridization between SBE and SQUen.

### 3.4 Genetic ancestry simulation for the hybrid lineage

Remarkably different allele frequencies between SBE and SQUen were found in eight of the 16 nuclear DNA loci (*17367, DNAH, FSHR, GHR, PRLR, TAR, TB62*, and *TB81*, Figure S6), which were considered species‐diagnostic loci to determine the genetic ancestry of SHY.

**FIGURE 6:**
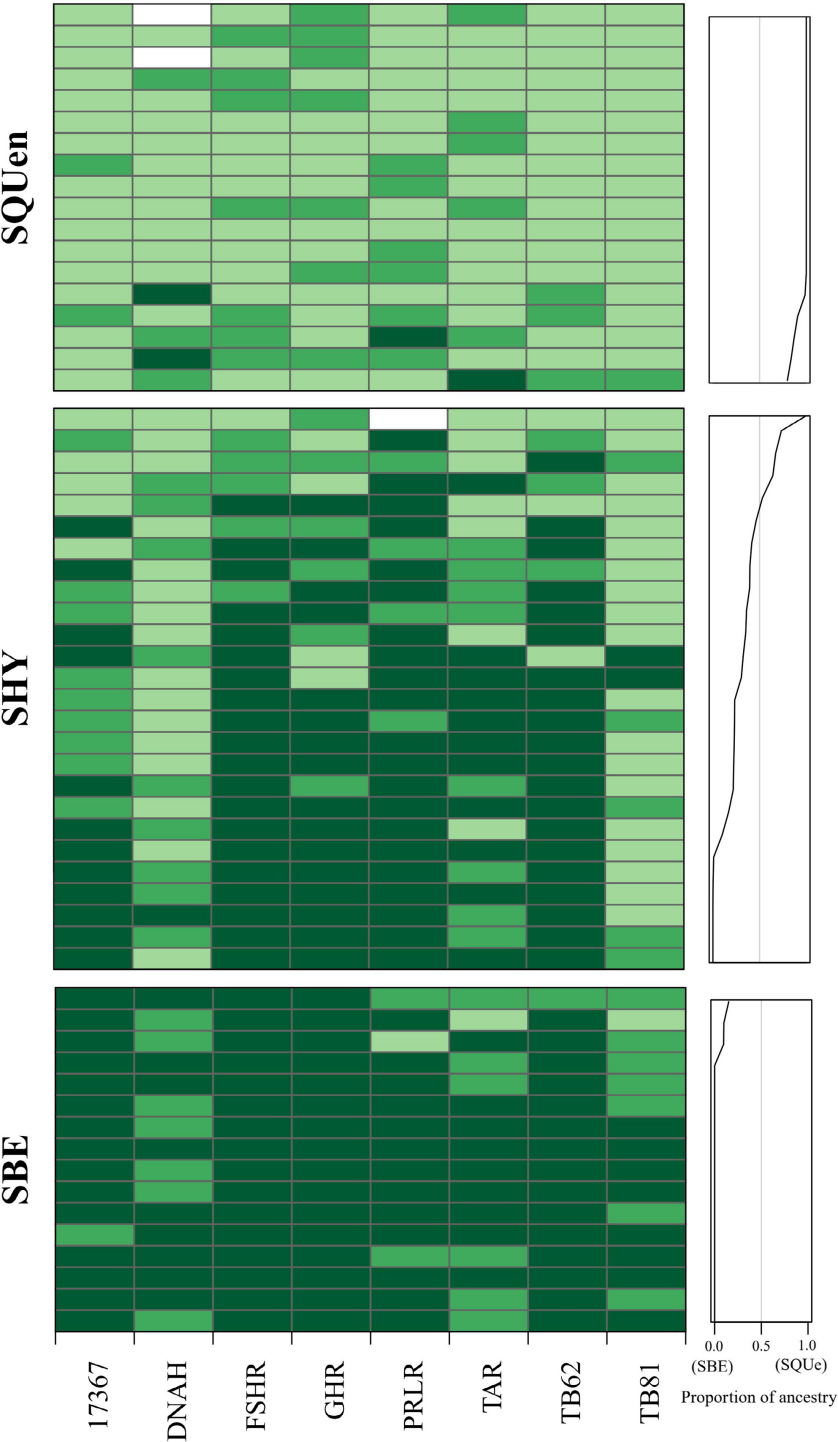
Ancestry analysis based on eight species‐diagnostic loci. Each row represents one individual. Dark green means that both alleles are nearly fixed in SBE, light green is for both alleles nearly fixed in SQUen and middle green indicates a combination of alleles nearly fixed in different populations. The estimated proportion of ancestry is plotted at the right of each individual.

Individuals in the SHY group showed admixed genetic ancestry composition in nuclear DNA markers (Figure 6). For *FSHR, GHR, PRLR*, and *TB62* loci, most individuals in SHY showed species‐diagnostic alleles of SBE. For *DNAH* and *TB81* loci, most alleles in SHY were diagnostic for SQUen. However, for *17367* and *TAR* loci, allele diagnostic for SBE and SQUen was almost equal to that for SHY.

In the simulation of hybridization between SBE and SQUen, all loci consisted of species‐equal diagnostic alleles of the two parental populations from the first to the 20th generation (5 years per generation). Unequal genetic ancestry composition was found among alleles of the 50th to 100th generations due to genetic drift, which was similar to the pattern observed for SHY (Figure S7). Complete fixation in all loci occurred between the 200th and 500th generations.

### 3.5 Morphological variations among different *Sacalia* populations

The morphological identification of different *Sacalia* species or geographical populations was majorly based on the dots and “eye” spots on the head, as well as spots or patches on the plastron (Table 5). Differences between *S. bealei* (SBE) and *S. quadriocellata* (SQU) were distinguishable. *S. quadriocellata* had no or only a few dots on its head and two pairs of eye‐like spots that were completely isolated and quite bright. *S. bealei* had dense black dots on top of the head, and the anterior eye‐like spots linked together with the posterior pair, making them rather obscure. The hybrid *Sacalia* turtles (SHY) showed mosaic and intermediate morphological characteristics between the two parental species, with the head looking more similar to *S. quadriocellata* but with darker eye‐like spots mostly similar to *S. bealei* (Figure 1b). The Hainan *S. quadriocellata* (SQUes) was also distinct from other *S. quadriocellata* subclades on the mainland in certain characteristics such as space between the posterior “eye” spots, spots on the chin, spots or patch density on the plastron, and smaller body size (Lin et al., 2018). Differences among SQUen and the three subclades of west *S. quadriocellata* have not yet been verified because of a lack of a sufficient number of specimens.

**TABLE 5:** Taxonomic delimitation of eyed turtles *Sacalia* sp. based on geographic distribution, population structure, and morphological characters.

	*S. bealei* (SBE)	*S. quadriocellata × bealei* (SHY)	Hainan *S. quadriocellata* (SQUes)	Northern east *S. quadriocellata*(SQUen)	Northern west *S. quadriocellata* (SQUwn)
Head	Have many black dots on the head. Two pairs of “eye” spots linked together while the first pair is obscure, especially in males	Have no dot or quite a few dots on the head. The “eye” spots look more similar to *S. quadriocellata*, but are darker and mostly linked together	Have no dot or quite a few dots on the head. The two pairs of “eye” spots are isolated and quite bright with white circles around them		
			The space between the posterior “eye” spots is wider and there are only some small scattered spots on the chin	The space between the eye spots usually forms a “V” posteriorly and there are two big spots on the chin	
Plastron	Have black spots (male) or patches (female) on plastron, but much fewer compared to other *Sacalia* turtles. Some individuals could have pure color without any spots or patches	Characters of spots or patches on plastron look more similar to *S. bealei*.	Most densely covered with black spots or patches	Covered with many black spots or patches	
Carapace Length (mm, Mean SE)	Female: 139.36 ± 2.04 (*n* = 40) Male: 127.82 ± 1.58 (*n* = 28)^a^	Female: 144.26 ± 5.97 (*n* = 10) Male: 126.00 ± 5.38 (*n* = 5)	Female: 132.0 ± 1.5 (*n* = 21) Male: 127.6 ± 1.2 (*n* = 20)^b^	130.83 ± 4.05 (*n* = 12)^c^	

^a^ Lin et al. (2017).

^b^ Xiao et al. (2014).

^c^ Lin et al. (2018).

Three individuals, which were morphologically identified as SQUen originating from Wuzhou, Guangxi, were genetically assigned as SHY, and one suspected hybrid individual was assigned to SQUen (Table S1). This indicated a low rate (~7.8%) of misidentification between the two populations based solely on the morphological characteristics of the eyespots. Further comparisons are required to identify more reliable morphological judgments.

## 4 DISCUSSION

### 4.1 Geographic population structure of *Sacalia*

The sympatric distribution of *S. quadriocellata* and *S. bealei* has been extensively documented in previous studies (Shi et al., 2013; Turtle Taxonomy Working Group, 2021). However, according to our long‐term survey and broad‐range sampling, we found that the two species exist exclusively in an area that is separated by the Pearl River drainage running across Guangxi and Guangdong Province (Figure 1). The Pearl River drainage is likely a natural barrier to dispersal as *Sacalia* turtles prefer small montane streams rather than large‐order rivers (Gong et al., 2006; Hu et al., 2016). A similar barrier effect of the Pearl River has also been revealed through a phylogeographic study of the black‐breasted leaf turtle *Geoemyda spengleri* (Gong, Chow, et al., 2009; Gong, Shi, et al., 2009). Therefore, previous reports of sympatric distribution are probably due to false identification of *S. bealei* and *S. quadriocellata*, which previously could not be differentiated (Shi, Fong, et al., 2008; Shi, Parham, et al., 2008), or due to individuals being moved via illegal trade.

The endemic population (SQUes) distributed on Hainan Island, separated from other populations by the Qiongzhou Strait, represents an extremely isolated population. Natural barriers, such as the Hai Van Pass, a portion of the Annamite Range and a well‐known biogeographic boundary in the country, may split the distribution of SQUwc and SQUws populations (Le et al., 2020). The boundaries that split east and west *S.quadriocellata* probably occur in the bordering area of western Guangxi and Northeastern Vietnam, which is a famous biodiversity hotspot area worldwide. The cause of the current distribution gap occurring in *S.bealei* is unclear, probably due to insufficient sampling effort, but they were probably isolated by natural or anthropogenic factors during population expansion. Further sampling efforts in this area are required to confirm this.

### 4.2 Natural hybrid origin of SHY

According to genetic analysis in this study, we believe that SHY is a near‐panmictic hybrid population and highlight its evolutionary potential of hybridization speciation. Various proportions of admixture in each individual (Figure 6) and near‐panmictic population properties (Figure 3 and Table 3) are characteristics of a long‐term persistent and self‐sustaining hybrid swarm (Li et al., 2016). However, the real population size is likely larger than the simulated 100 individuals per generation, which ensures that they can escape from stochastic extinction. Furthermore, long‐lasting backcross with parental populations would slow the genetic drift, which is common in hybrid swarms. Therefore, the actual history of SHY is likely to be much longer and more complicated than the simulation (100 generations). All mtDNA samples of SHY were from northern east *S. quadriocellata*, indicating that hybridization was strongly sex‐biased or the mtDNA from *S. bealei* was eliminated during the post‐isolation genetic drift.

The discovery of SHY represents the first report of a natural hybrid population of wild turtles in China. The last report of wild hybrid turtles was “*Cuora serrata*”‐like turtles in Hainan, which originated from independent hybrid events between male *C. mouhotii* and female *C. galbinifrions* (Shi et al., 2005). More than a dozen other newly described turtle species from China are artificial hybrids, and no wild specimens have ever been found (Parham et al., 2001; Stuart & Parham, 2007). The existence of the SHY population in the wild indicates that the natural hybridization of turtles has existed for a long time and has formed a distinct population. Recent studies have reported growing evidence that hybridization among distinct animal species contributes to gene pools of evolving lineages and may result in the formation of new species to an unprecedented extent (Capblancq et al., 2015; Vamberger et al., 2017). The near‐panmictic state of SHY is common at the beginning of hybrid speciation. However, we did not investigate whether it has distinct adaptive genetic, morphological, or ecological characteristics, which illuminates the progress of hybrid speciation. Precise dating of origin, mechanism of sex‐biased admixture, and evolutionary adaptation of this population require further genomic study.

### 4.3 Taxonomy

It is recommended to delimit species, publish new species, and conduct taxonomic revisions following certain conditions and different approaches (Liu, 2016). From the perspective of taxonomic operability, on the premise of sufficient phylogenetic evidence, the discontinuity or statistical discontinuity of two or more independent morphological traits between populations could be used as the preferred basis for judging species status, so that others can readily distinguish species (Hong, 2016; Liu et al., 2019).

For Hainan *S. quadriocellata* (SQUes), phylogenetic analysis showed that it is a distinct lineage and the most isolated population, which is consistent with a previous study (Lin et al., 2020). Our previous study on geometric morphometrics revealed that the body size, shape of the head and shell, and the number of spots or the ratio of the relative patch area on the plastron all showed significant differences among individuals between Hainan and mainland China (Lin et al., 2018; Sun, 2015). In addition, Hainan *S. quadriocellata* is geographically isolated from other populations and meets the conditions of reproductive isolation and ecological niche differentiation. Therefore, the consideration of Hainan *S. quadriocellata* as a “good species,” meets the conditions of multiple species concepts, and we support its status as an independent species named Hainan Four‐eyed Turtle (*S. insulensis*). However, this promotion causes that the four remaining subclades in *S. quadriocellata* are mitochondrially paraphyletic. Elevation of each subclade to species may be advisable based on more solid genomic and morphological evidence, but present limited research hinders comprehensive taxonomic delimitation of *S. quadriocellata* complex. Considering the diagnosable distinctiveness of both morphological and genetic characteristics, SHY conforms to the definition of hybrid taxon as “an independently evolving, historically stable population or group of populations possessing a unique combination of heritable characteristics derived from two or more discrete parental taxa” (Allendorf et al., 2001). Therefore, SHY should be treated separately as an independent natural hybrid taxon, *S. quadriocellata* × *bealei*. To facilitate dissemination and later discussion, we recommend appropriate common names for SHY and the other four lineages of *S. quadriocellata* according to their distribution range. Since SHY was first discovered in Zhaoqing, Guangdong, we suggest naming it the Zhaoqing Four‐eyed Turtle, SQUen the South Chinese Four‐eyed Turtle, SQUwn the Northern Vietnamese Four‐eyed Turtle, SQUwc the Central Vietnamese Four‐eyed Turtle, and SQUws the Southern Vietnamese Four‐eyed Turtle.

### 4.4 Conservation implications

Like many other Asian freshwater turtle species, poaching has resulted in dramatically reduced populations of *Sacalia* turtles, even in nature reserves (Gong et al., 2017; Hu et al., 2016). Illegal trade including long‐distance and cross‐border transportation is also common for *Sacalia* turtles. Here, some *S. quadriocellata* individuals found in Guangdong province belonged to the SQUwn subclade in northern Vietnam, and those in the Qingzhou City of Guangxi originated from SQUwc and SQUws subclades in central Vietnam (Table S1). The long‐term absence of legal protection and weak law enforcement are mainly responsible for the turtle survival crisis (Wang et al., 2021). In China, for example, *S. bealei* and *S. quadriocellata* were not included on the “List of Wildlife under Special State Protection” until 2021, and even then, only wild populations were under protection. This is inadequate and difficult for conservation management and implementation, as wild and captively bred individuals are difficult to distinguish unless identified by molecular measures. Furthermore, it is common to mix different geographical populations and even different species through husbandry and captive breeding of *Sacalia* turtles in China, which can easily lead to genetic contamination and is not conducive for future reintroduction into the wild. Therefore, more powerful enforcement and precise management should be applied to both ex situ and in situ conservation. Since the artificial breeding of *Sacalia* turtles is still immature, studies have shown that turtle farms are the primary purchasers of wild‐caught turtles and lucrative farming operations are a major threat to the survival of China's diverse turtle fauna (Shi et al., 2007). We recommend that the government shall discourage captive breeding of *Sacalia* turtles, and the “List of Wildlife under Special State Protection in China” does not emphasize that only wild populations are protected. This recommendation also applies to many other endangered freshwater turtles in Asia, such as the genus *Cuora* and *Platysternon megacephalum*.

Despite the difficulties of species delimitation for the *S. quadriocellata* complex and SHY, we suggest that the six subclades and the near‐panmictic hybrid population should be recognized as seven different evolutionarily significant units. The conservation of hybrids is controversial. However, we justify the eligibility of separate conservation of SHY based on its stable population genetic distinctiveness, which represents natural genetic diversity and evolutionary potential of hybrid speciation in the genus *Sacalia*. A narrow‐ranged population tends to be seriously threatened by overharvesting of the two endangered parental species (Allendorf et al., 2001; Jackiw et al., 2015). Phylogeographic and morphological differentiation needs to be addressed in husbandry and captive management to avoid lineage admixture and to preserve their genetic diversity, especially SHY and SQUes, which inhabit quite a small range.

## 5 CONCLUSION

In this study, we provide a comprehensive understanding of genetic diversity, geographical distribution, and gene flow among the populations of the genus *Sacalia*. Six mitochondrial subclades were found within this genus, indicating a higher diversity than expected. The “hybrid‐eyed turtle” lineage represents a natural panmixia derived from hybridization between *S. bealei* and *S. quadriocellata*, and it is also the first report of a natural hybrid turtle population in China. Taken together, *Sacalia* lineages should be considered as seven different evolutionarily significant units and admixture in captive breeding should be avoided. However, a comprehensive taxonomy of *S. quadriocellata* complex requires further morphological and genomic data. More sampling efforts should be directed to some specific areas, such as the *S. bealei* range and the western part of Guangxi , to verify the distribution pattern. The fine‐scale sampling in this study demonstrates the rich genetic diversity of eyed turtles in South China, which sheds light on the vast cryptic diversity of freshwater turtles in Asia and highlights the urgency for further herpetological research and conservation in this region.

## AUTHOR CONTRIBUTIONS

**Liu Lin:** Conceptualization (lead); data curatinon (lead); formal analysis (lead); funding acquisition (lead); investigation (lead); methodology (lead); project administration (lead); resources (lead); software (equal);validation (lead); writing–original draft (lead); writing–review and editing (lead). **Huaiqing Chen:** Conceptualization (lead); data curation (lead); formal analysis (lead); funding acquisition (equal); investigation (lead); methodology (lead); project administration (lead); resources (equal); software (lead); validation (lead); writing – original draft (lead); writing – review and editing (lead). **Daniel Gaillard:** Conceptualization (equal); data curation (supporting); formal analysis (equal); funding acquisition (supporting); investigation (equal); methodology (equal); resources (equal); software (supporting); visualization (equal); writing – original draft (equal); writing – review and editing (equal). **Hai‐Tao Shi:** Conceptualization (lead); data curatinon (supporting); formal analysis (equal); funding acquisition (lead); investigation (equal); project administration (lead); resources (lead); supervision (lead); writing‐original draft(equal); writing‐review and editing (equal). **Shu‐Jin Luo:** Conceptualization (equal); data curation (supporting); formal analysis (equal); funding acquisition (equal); project administration (equal); resources (equal); supervision (lead); writing – original draft (equal); writing – review and editing (equal).

## 6 FUNDING INFORMATION

This study was supported by the National Nature Science Foundation of China (31960101, 32170532, 32070598) and the Natural Science Foundation of Hainan Province (319MS048). The funders had no role in the study design, data collection and analysis, decision to publish, or preparation of the manuscript.

## Supporting information


Table S1.

Table S2.

Table S3.

Table S4.

Table S5.

Figure S1.

Figure S2.

Figure S3.

Figure S4.

Figure S5.

Figure S6.

Figure S7.

Figure S8.

## Data Availability

All genetic sequences were submitted to GenBank. The accession number of mitochondrial gene sequences is OP908986‐OP909016. See Table S3 for the accession numbers of nuclear gene sequences.
