# Supplementary figures and images for: Phylogeography of the endangered “eyed” turtles (genus Sacalia) and the discovery of a lineage derived from natural interspecific hybridization
Source: Ecol Evol. 2022 Dec 21;12(12):e9545. doi: 10.1002/ece3.9545 (PMC9772493; doi:10.1002/ece3.9545)

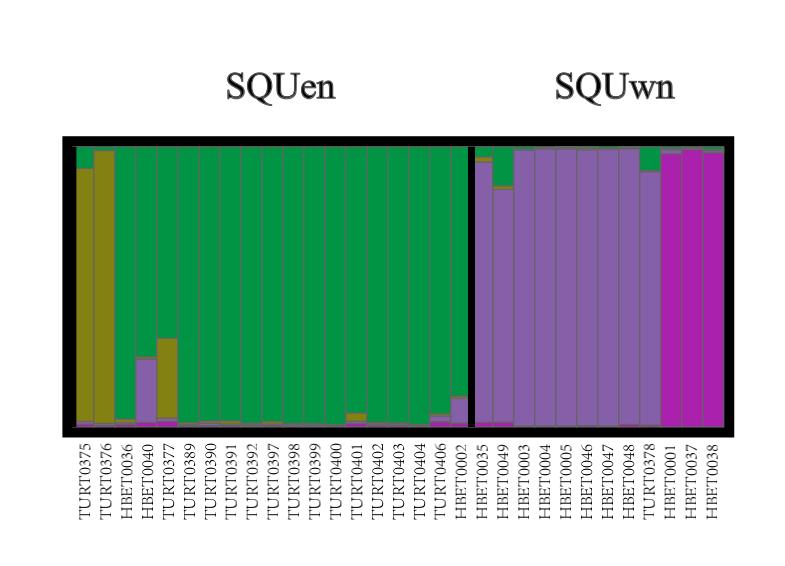

Supplement: Supplementary file 6 — Figure S1. [file ECE3-12-e9545-s003.jpg]

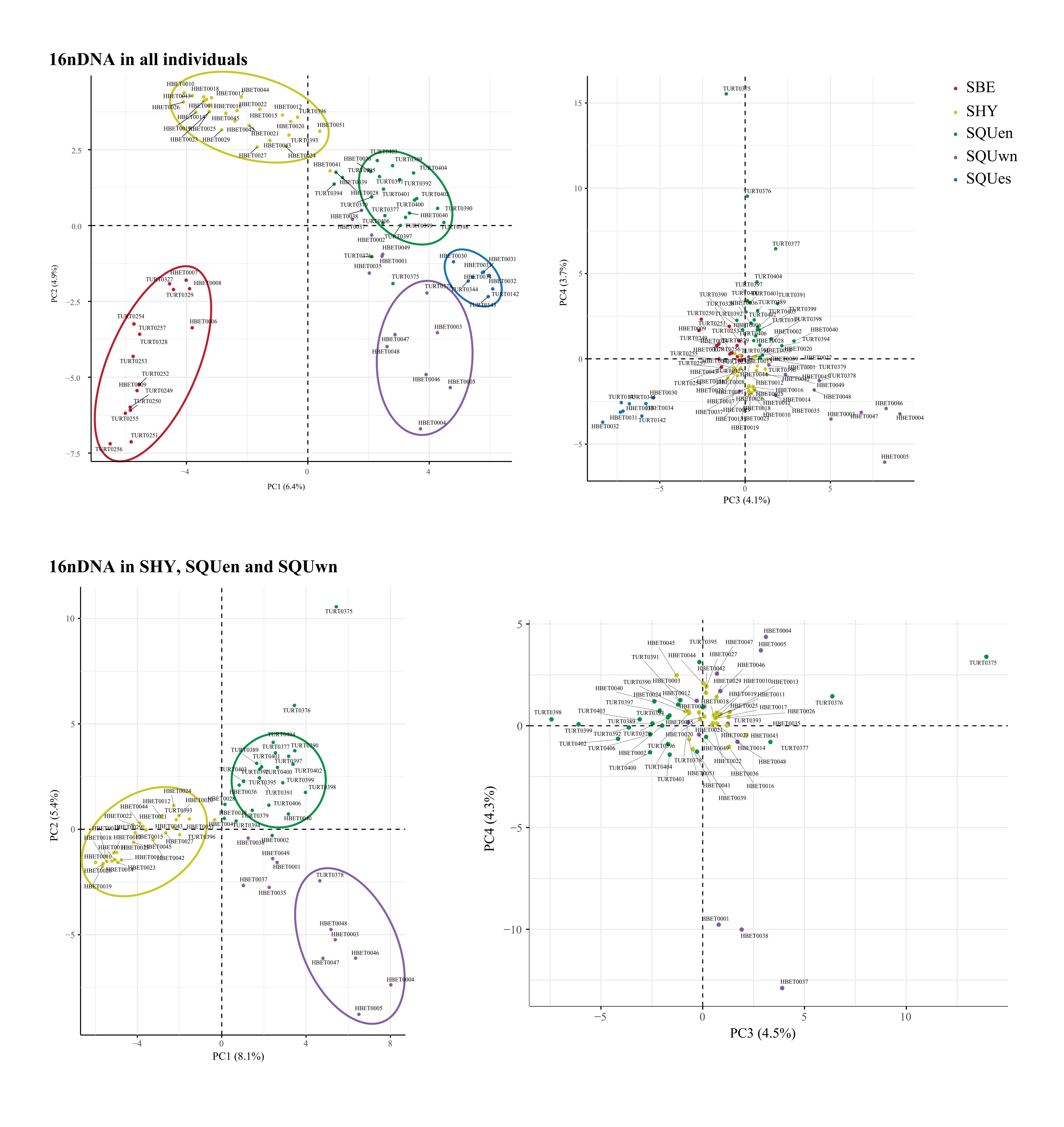

Supplement: Supplementary file 7 — Figure S2. [file ECE3-12-e9545-s013.jpg]

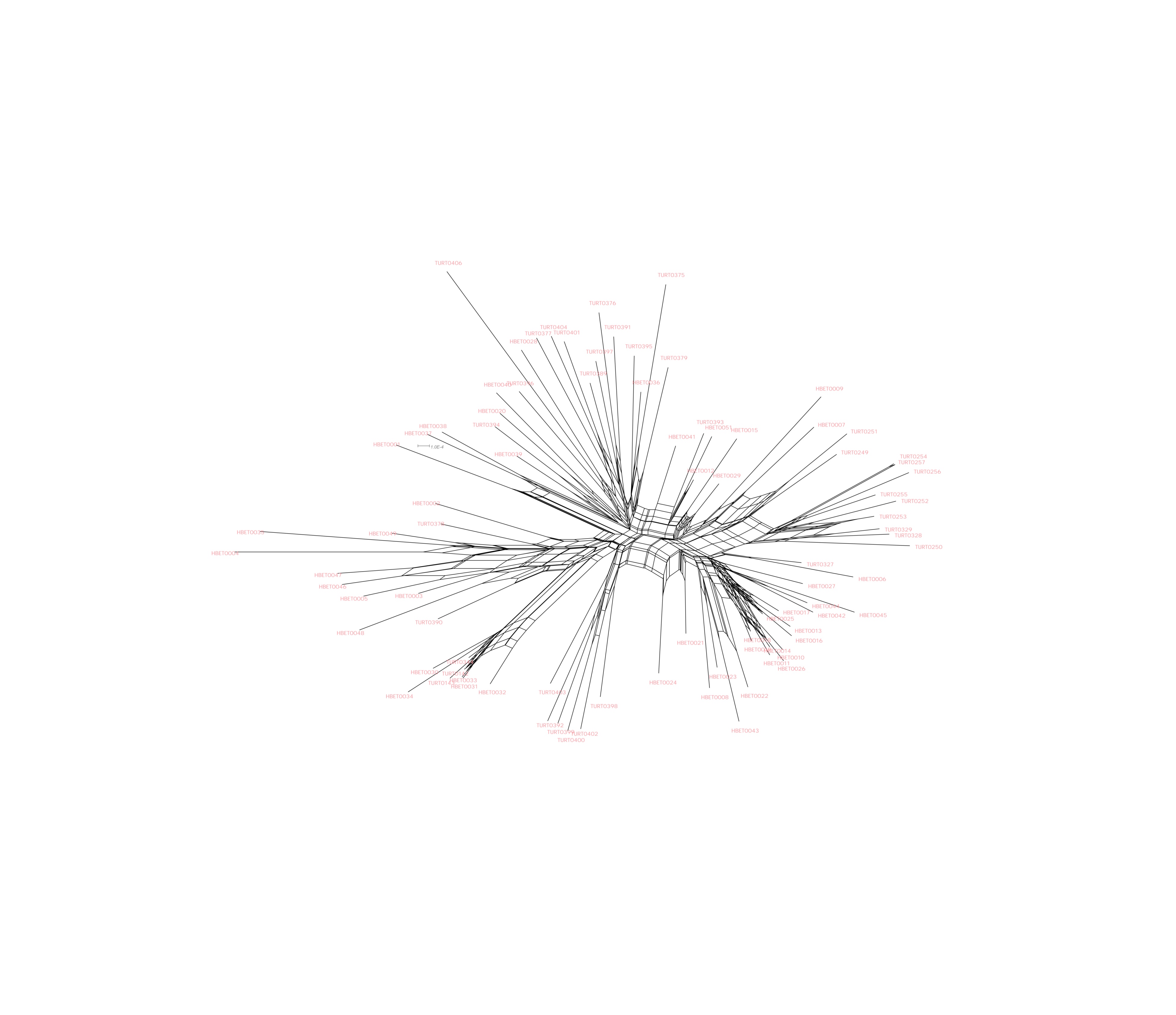

Supplement: Supplementary file 8 — Figure S3. [file ECE3-12-e9545-s009.jpg]

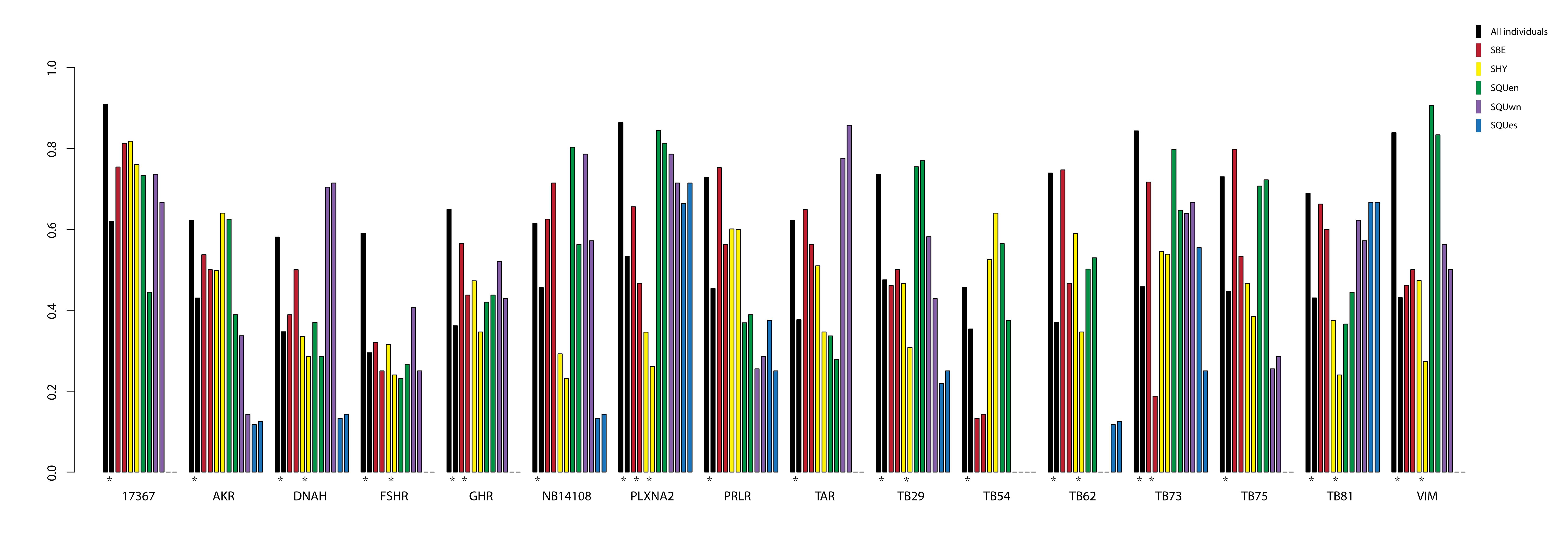

Supplement: Supplementary file 9 — Figure S4. [file ECE3-12-e9545-s008.jpg]

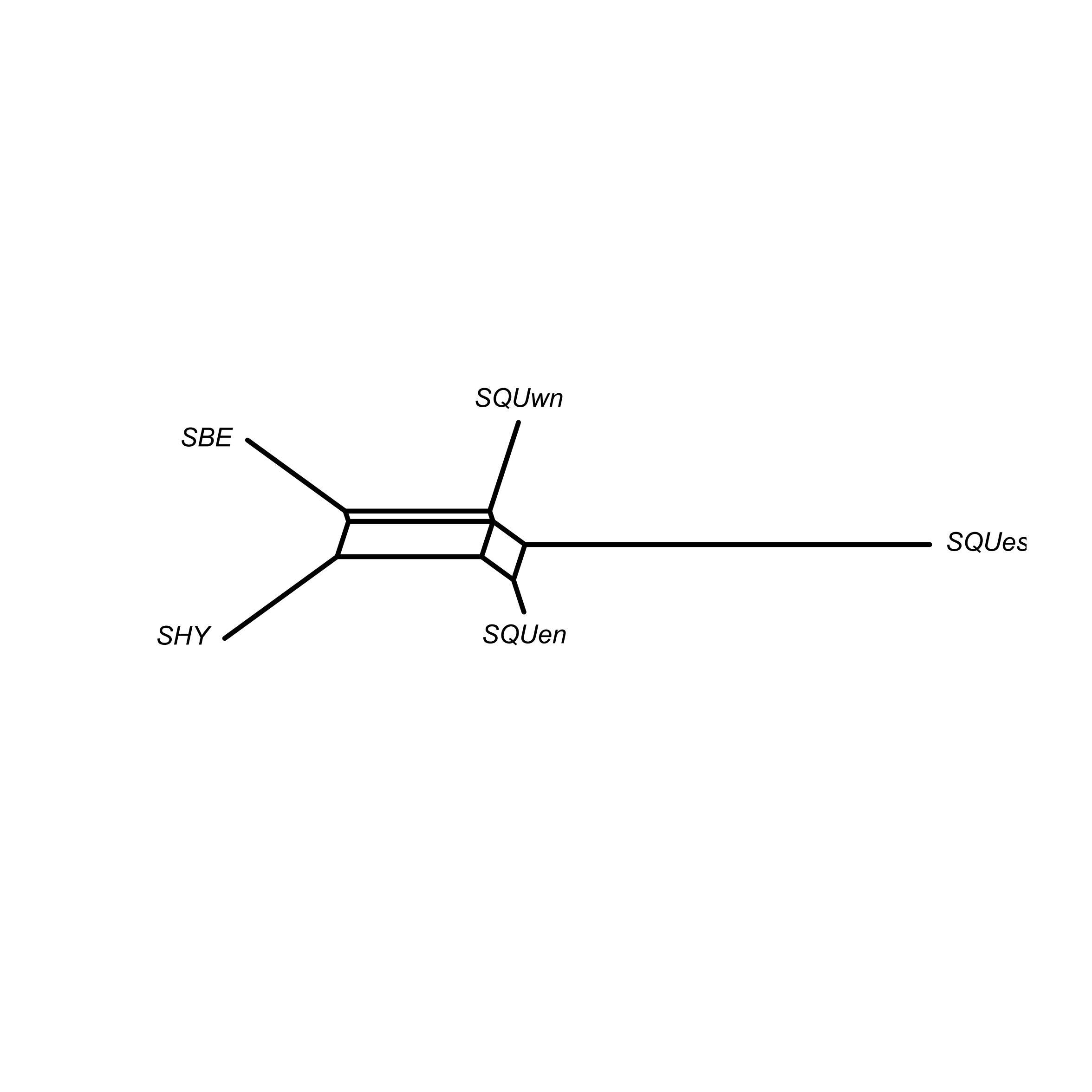

Supplement: Supplementary file 10 — Figure S5. [file ECE3-12-e9545-s012.jpeg]

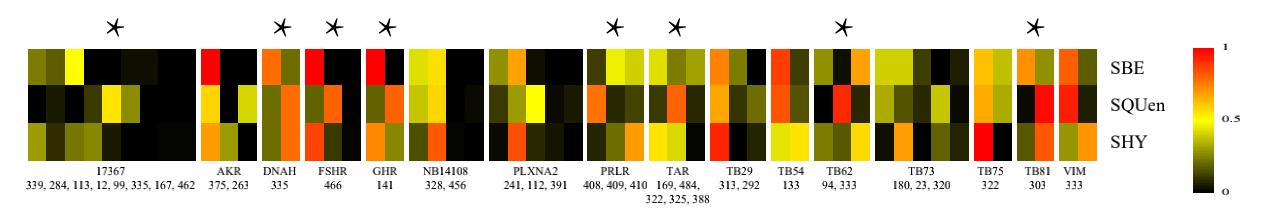

Supplement: Supplementary file 11 — Figure S6. [file ECE3-12-e9545-s007.jpg]

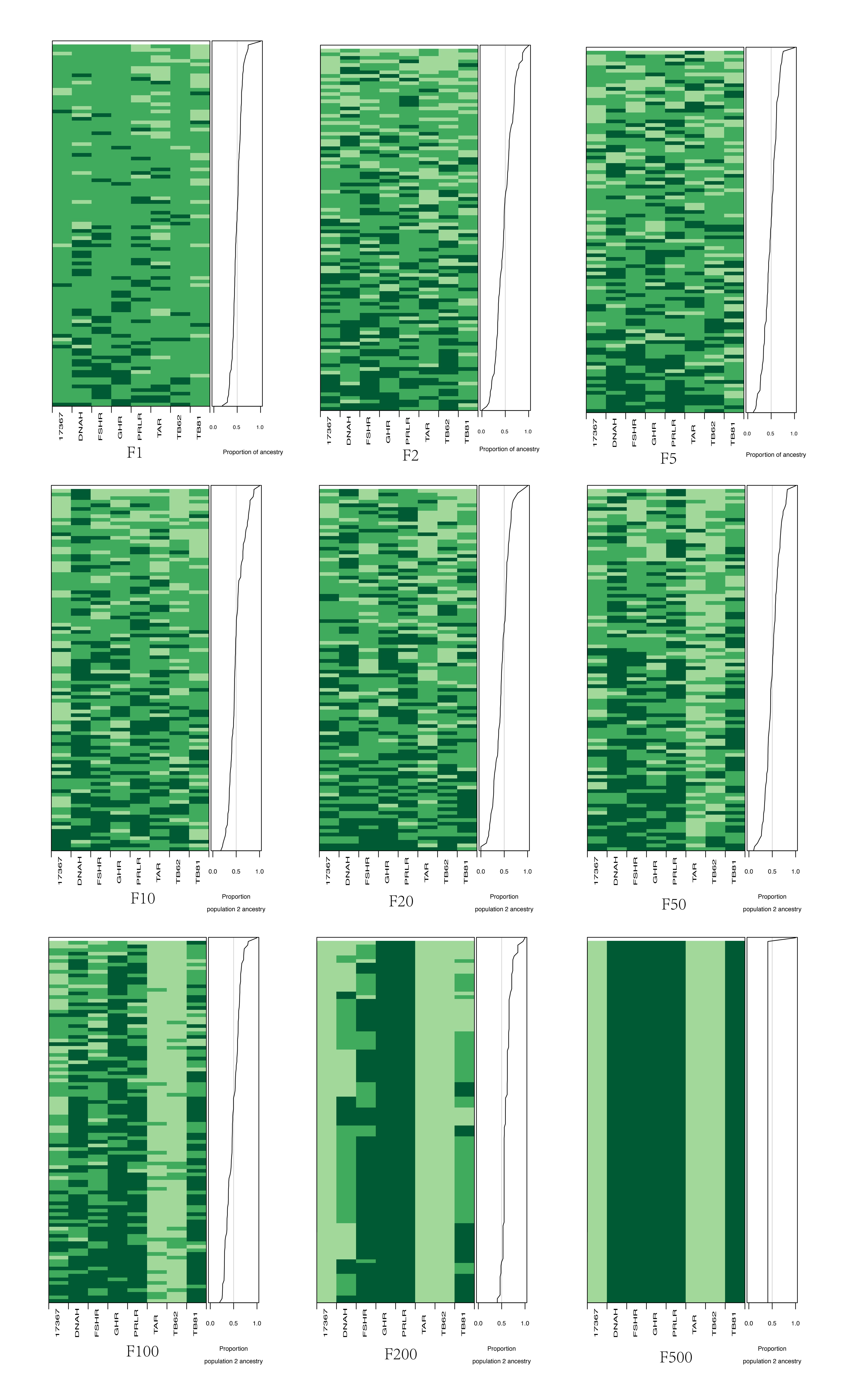

Supplement: Supplementary file 12 — Figure S7. [file ECE3-12-e9545-s006.jpg]

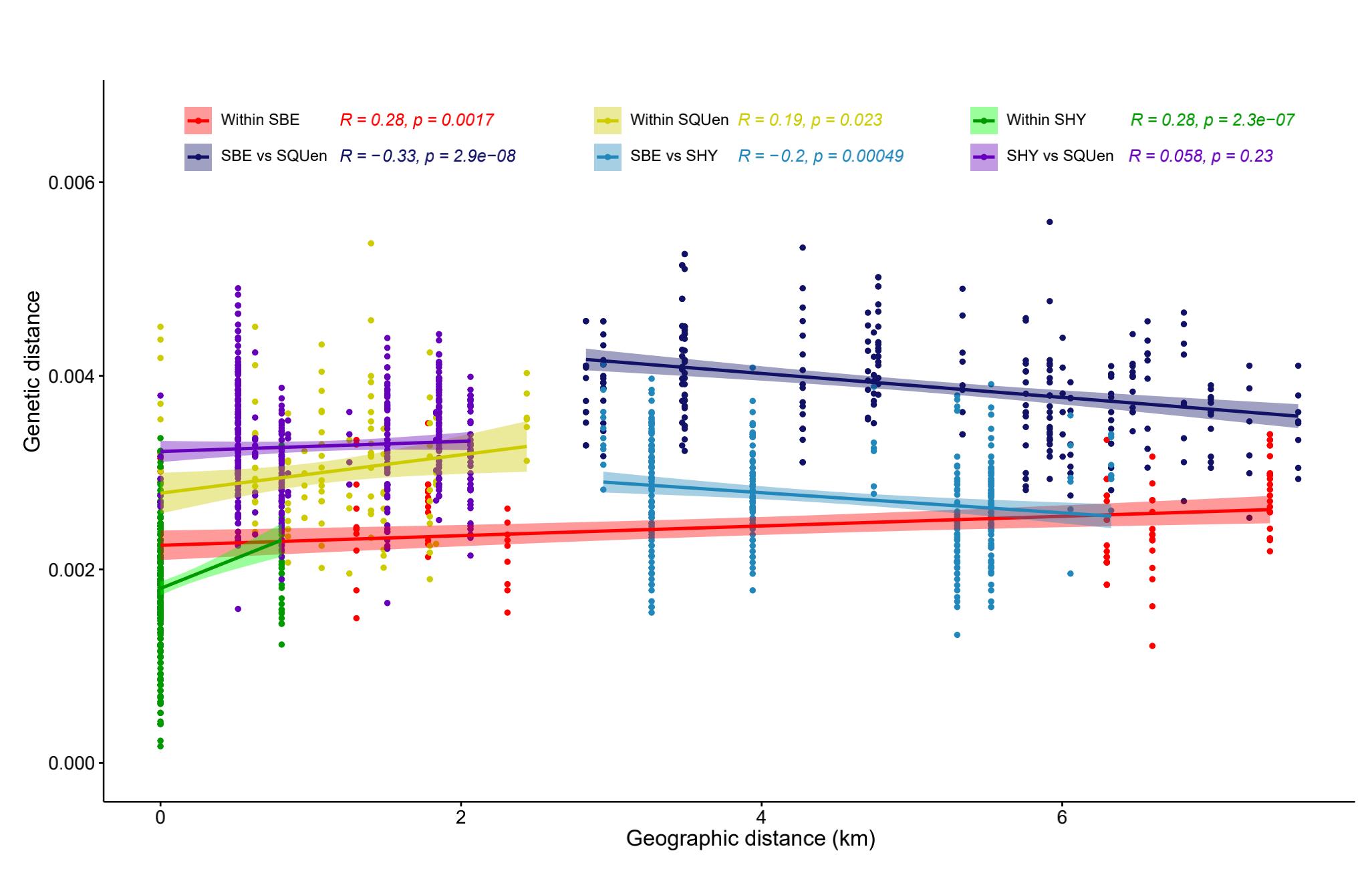

Supplement: Supplementary file 13 — Figure S8. [file ECE3-12-e9545-s011.jpg]
